# Back-projection of COVID-19 diagnosis counts to assess infection incidence and control measures: analysis of Australian data

**DOI:** 10.1017/S0950268820001065

**Published:** 2020-05-18

**Authors:** I. C. Marschner

**Affiliations:** National Health and Medical Research Council Clinical Trials Centre, University of Sydney, Sydney, New South Wales, Australia

**Keywords:** Coronavirus, COVID-19, epidemiology, statistics, surveillance

## Abstract

Back-projection is an epidemiological analysis method that was developed to estimate HIV incidence using surveillance data on AIDS diagnoses. It was used extensively during the 1990s for this purpose as well as in other epidemiological contexts. Surveillance data on COVID-19 diagnoses can be analysed by the method of back-projection using information about the probability distribution of the time between infection and diagnosis, which is primarily determined by the incubation period. This paper demonstrates the value of such analyses using daily diagnoses from Australia. It is shown how back-projection can be used to assess the pattern of COVID-19 infection incidence over time and to assess the impact of control measures by investigating their temporal association with changes in incidence patterns. For Australia, these analyses reveal that peak infection incidence coincided with the introduction of border closures and social distancing restrictions, while the introduction of subsequent social distancing measures coincided with a continuing decline in incidence to very low levels. These associations were not directly discernible from the daily diagnosis counts, which continued to increase after the first stage of control measures. It is estimated that a one week delay in peak incidence would have led to a fivefold increase in total infections. Furthermore, at the height of the outbreak, half to three-quarters of all infections remained undiagnosed. Automated data analytics of routinely collected surveillance data are a valuable monitoring tool for the COVID-19 pandemic and may be useful for calibrating transmission dynamics models.

## Introduction

As the COVID-19 pandemic has evolved, daily counts of new diagnoses have been a major focus of governments, researchers and the broader community. However, although these counts provide a window into the progress of the epidemic, they represent only part of the total extent of infection within a population. Furthermore, the pattern of diagnoses over time is a lagged and incomplete representation of the pattern of infections over time. In monitoring the extent and evolution of the epidemic, as well as the effectiveness of control measures such as border closures, social distancing and community lockdowns, it is infections not diagnoses that are of primary interest.

The purpose of this paper is to analyse Australian COVID-19 daily diagnosis counts in order to demonstrate how these routinely collected data can be converted into information about the quantity of fundamental interest, which is infection incidence. It is demonstrated that with appropriate statistical analysis, based on back-projection methodology used extensively to monitor the HIV/AIDS epidemic, the daily COVID-19 diagnosis data can provide information about serial infection incidence and the extent of undiagnosed infections in a population. As well as helping to understand the extent of the epidemic, such information can also be used to assess the impact of government control measures. It is argued that analytics systems automating and continuously updating COVID-19 infection estimates and projections are valuable tools for monitoring the outbreak, and should be considered as preparation for a potential second wave of infections.

## Methods

### Diagnosis data

In Australia, each of the eight state and territory health departments provides a daily update on the cumulative number of COVID-19 diagnoses. Various public domain resources provide convenient access to these data [1–5]. This paper uses confirmed case numbers taken directly from the daily health department updates. Daily diagnosis numbers were analysed for the 105 days from the first reported case on 25 January through to 8 May, broken down by state and territory as well as aggregated nationally. Although slight differences exist between the various public domain data resources, the analysis results were not substantively altered when repeated on other versions of the data.

### Estimating infection incidence

The primary method of analysis was the method of back-projection, also called back-calculation, which was originally developed for the purpose of estimating HIV incidence using AIDS surveillance data [6–8]. This method has also been used extensively in other contexts where an unobserved incident event is followed by a delay until an observed diagnosis [9, Section 3.1], as is the case for COVID-19 infection and diagnosis.

A key piece of information required to implement a back-projection analysis is the probability distribution of the time *D* between the unobserved event of interest and the subsequently observed diagnosis, which will be referred to as the diagnosis distribution. The diagnosis distribution specifies the probabilities 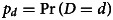 for a given number of days *d*, and will be described in detail below. Back-projection then makes use of the fact that the observed serial diagnosis counts *Y*_*t*_, for days *t* = 1, … , *T*, reflect the unobserved number of COVID-19 infections *X*_*t*_, aggregated with the diagnosis distribution. Given the assumed diagnosis distribution, these diagnosis counts can be statistically decomposed to yield estimates of the infection incidence, from which estimates of cumulative infections and undiagnosed infections may also be obtained.

The analysis is based on the fundamental relationship between the mean diagnosis count at time *t*, *μ*_*t*_ = *E*(*Y*_*t*_), and the mean infection counts *λ*_*s*_ = *E*(*X*_*s*_), for times *s* up to and including time *t*, weighted by the diagnosis distribution:
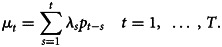
Assuming that the infection counts *X*_*t*_ are independent Poisson random variables leads to a high-dimensional linear Poisson regression model for the diagnosis counts *Y*_*t*_. The model requires non-negativity constraints on the parameters *λ*_*t*_, reflecting the fact that they are Poisson means. Once fitted, the model provides estimates of the mean infection incidence 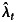, which can be used as estimates of the unobserved infection counts over time. This process is called back-projection because it effectively involves projecting the diagnosis counts back in time using the diagnosis distribution, and is also referred to as Poisson deconvolution because it involves disaggregating the mean infection counts from the diagnosis distribution. In theory, the model is an identity link generalised linear model, but in practice it requires special computational software to accommodate the high dimensionality and the parameter constraints. Reliable algorithms for this non-standard analysis are incorporated into freely available open source software and are used here. In particular, the analyses were performed in the R computing environment [10] using the addreg package that contains the nnpois function for performing reliable high-dimensional non-negatively constrained linear Poisson regression [11]. Code for implementing these analyses is available on the GitHub repository linked to the Coronavirus 10-day Forecast resource from The University of Melbourne [4].

The back-projection estimates 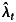 provide information about the overall incidence of infection among the population presenting for diagnosis. Importantly, back-projection does not estimate the incidence of community transmission, because it includes both infections derived from community transmission as well as those imported from outside the population. Instead, back-projection provides an assessment of the incidence of infections aggregated over all sources, which can then be used to provide information about the effectiveness of control measures and the pattern of future case diagnoses. Conversely, transmission models incorporate the dynamics of different sources of infection making up the aggregated infection incidence, and for this reason back-projection is useful for calibrating transmission models.

In view of the high dimensionality of the model, the analysis additionally makes use of smoothing, which was implemented using a simple moving average of width one week. This smoothing was applied first to the daily diagnosis counts, and then in each iteration of the computational algorithm for fitting the high-dimensional linear Poisson model [12, 13]. Confidence intervals were obtained using parametric bootstrap methods [6]. A total of 1000 bootstrap replications of the diagnosis data were generated and the back-projection analysis was applied to each, after which the 2.5% and 97.5% percentiles of the incidence estimates were used as 95% confidence intervals. Adjustment for over-dispersion relative to the Poisson model was incorporated by generating replications from a negative binomial distribution with mean equal to the model fitted values and variance inflated by the over-dispersion factor [6, p. 199]. As in previous smoothed back-projection analyses, the model fitted values for generating bootstrap replications were computed using an unsmoothed back-projection analysis [13]. As described earlier in this section, all data and software for implementing these analyses is available in the public domain.

### Incubation, testing and diagnosis periods

**Fig. 1.**
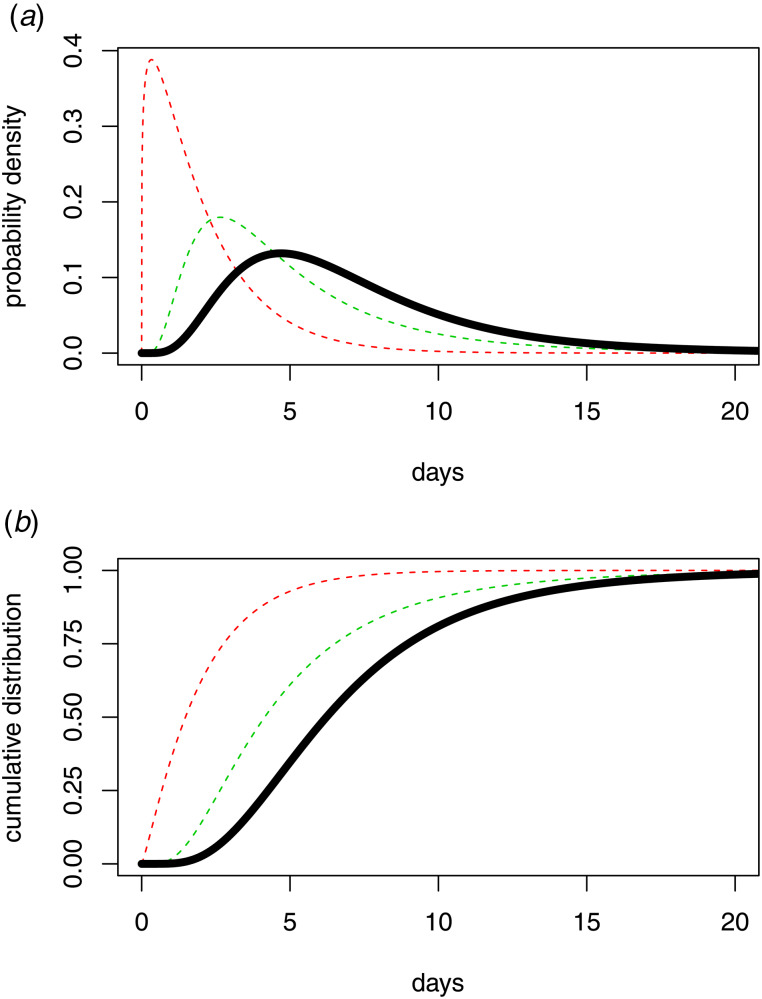
Distribution of the time between COVID-19 infection and diagnosis (black lines), which is the aggregate (convolution) of the incubation distribution (green lines) and the distribution of the time between symptoms and diagnosis (red lines). Panel A plots the probability density functions and panel B plots the cumulative distribution functions.

As introduced above, estimation of infection incidence using back-projection requires an assumed probability distribution *p*_*d*_ for the number of days *D* between infection and diagnosis. For COVID-19, there are two primary phases contributing to this diagnosis distribution: the incubation period between infection and the development of symptoms, and the testing period between symptoms and final diagnosis. These two components of the diagnosis distribution will be referred to as the incubation distribution and the testing distribution, respectively. Information about the incubation distribution has been published [14] and has been incorporated into transmission modelling used by the Australian government [15, 16]. Based on this information, the analysis presented here used a log-normal incubation distribution with an average of 5.2 days and a 95% percentile of 12.5 days [14]. Likewise, modelling used by the Australian government has assumed a mean testing period of 2 days [16], which is reflected here using a gamma distribution with rate parameter 0.6 per day and shape parameter 1.2. This gamma distribution was chosen so as to closely approximate an exponential distribution with mean 2 days, but with zero probability density at the time origin. Using the incubation and testing distributions, and assuming that the two periods are statistically independent, the diagnosis distribution is the probabilistic convolution of the two distributions. Figure 1 plots the probability density function *f*(*d*) and the cumulative distribution function *F*(*d*) for the diagnosis period *D*, as well as the underlying incubation and testing distributions. The delay between infection and diagnosis is consequently assumed to have a mean of 7.2 days and 95% percentile of 15.1 days. To facilitate the linear Poisson regression analysis of discrete-time daily data, the diagnosis distribution is represented as a discretised version of the continuous-time distribution:
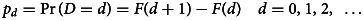
Since misspecification of the diagnosis distribution can bias the infection incidence estimates, sensitivity analyses were conducted to assess the robustness of the primary conclusions. These were conducted by repeating the back-projection analysis with both short and long diagnosis distributions. The short distribution used an incubation distribution with mean 4.1 days (lower 95% confidence limit reported in [14]), combined with a testing distribution having double the rate of diagnosis and therefore half the mean time from symptoms to diagnosis. The long diagnosis distribution was constructed similarly, with mean incubation period 7.0 days (upper 95% confidence limit) and half the rate of diagnosis or double the mean time from symptoms to diagnosis. The sensitivity analyses therefore had mean diagnosis periods from 5.1 to 11.0 days, compared to the assumed mean of 7.2 days.

### Undetected infections

An implicit assumption of the presentation of the diagnosis distribution is that it is a proper distribution in the sense that it sums to 1. In practice this may not be the case because some infected individuals may remain undetected by the testing regime, particularly those with asymptomatic infection. In this case the diagnosis distribution is a sub-distribution, in the sense that
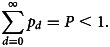
Here *P* is the proportion of infections that are detected by the testing regime, and 1 − *P* is the proportion of undetected infections. If *P* < 1 then the diagnosis distribution has cumulative distribution function *P* × *F*(*d*), which means that the linear Poisson model is re-scaled by a factor *P*. The consequence of this re-scaling is that, in the presence of undetected infections, the back-projection estimates 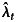 are estimates of *P* × *λ*_*t*_ rather than the actual infection incidence *λ*_*t*_ [6, pp. 201–202]. Thus, back-projection applied to diagnosis counts may produce under-estimates of infection incidence, particularly if the extent of undetected infections is substantial.

Despite the potential for under-estimation, there are two key points that make back-projection estimates useful even in the presence of undetected infections. Firstly, it is possible to make a range of assumptions about *P* or to estimate it from hospitalisation data, and then to inflate the back-projection estimates by a factor of 1/*P* in order to adjust for undetected infections [16–18]. For example, the COVID-19 transmission model used by the Australian government [16], as well as other published models [19], incorporate assumptions about the net proportion of cases that present for diagnosis, which corresponds to *P*, and which can be used straightforwardly to adjust the estimates for undetected infections. One published estimate of *P* used by the Australian government is 0.93, which would suggest that the infection incidence estimates need to be inflated by 7.5% [17, 18]. Secondly, assuming that *P* is relatively stable over time, or in other words that the testing regime is relatively stable over time, the shape of the infection incidence curve will be unchanged in the presence of undetected infections. In this case, qualitative features of back-projection estimates, such as the timing of peak incidence and its temporal association with control measures, are unaffected by the existence of undetected infections.

### Control measures

Estimates of infection incidence were compared with the timing of key government control measures. Following early measures such as limiting outdoor and indoor gathering sizes to hundreds, the Australian government implemented staged restrictions during the period 20 March to 31 March [20]. The initial stage involved border closures (20 March) and Stage 1 social distancing restrictions (23 March) including prohibition of many types of face-to-face business and entertainment activities. These were followed by Stages 2 and 3 social distancing restrictions (26–31 March), which included limiting gatherings to two people and restrictions on leaving the home except for essential purposes.

## Results

### Infection diagnosis

**Fig. 2.**
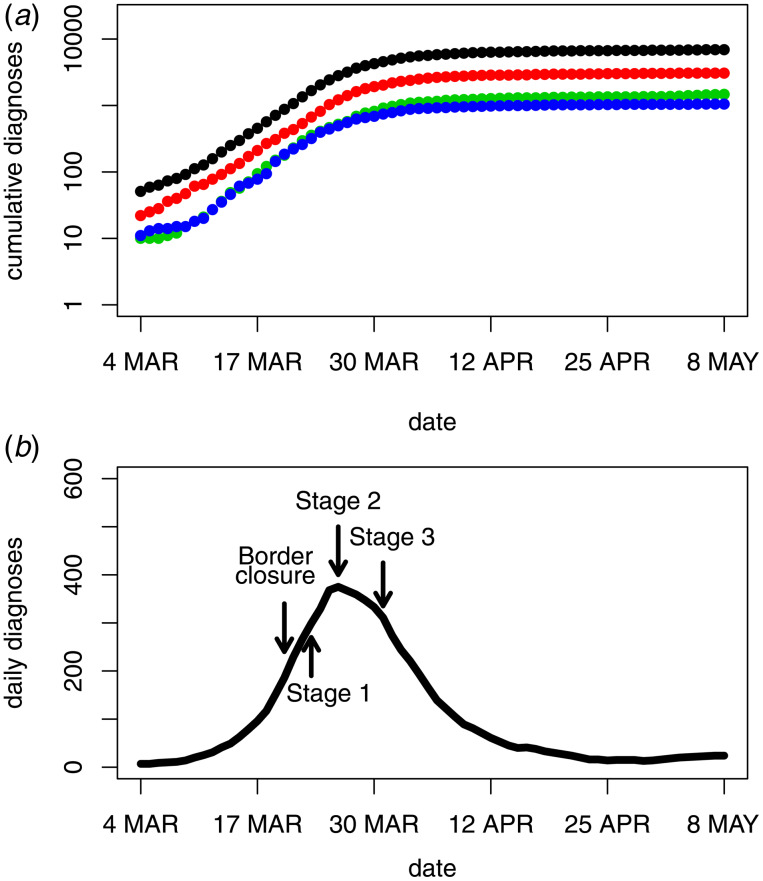
Daily COVID-19 diagnosis counts for Australia. Panel A plots the cumulative counts (log scale) for Australia (black) and the three states with the largest outbreaks, New South Wales (red), Victoria (green) and Queensland (blue). Panel B plots the smoothed daily number of new cases for Australia along with the timing of government restrictions.

The cumulative daily diagnosis counts for Australia are presented in Figure 2 (panel A) for the period subsequent to the national total reaching 50 cases. As at 8 May, 6943 diagnoses were reported nationally. In addition, data from the three states with the largest outbreaks, comprising over 80% of the total epidemic, are also provided. By viewing the diagnosis counts on the logarithmic scale, it is seen that the growth of the epidemics in each state followed a similar pattern to the national trajectory. The smoothed daily national diagnosis counts are also shown in Figure 2 (panel B), together with the timing of key national government control measures. With respect to the pattern of daily diagnoses, border closures and Stage 1 restrictions were accompanied by a continued increase in the incidence of confirmed cases, whereas Stages 2 and 3 restrictions were accompanied by a peak and decline in incidence of new case diagnoses.

### Infection incidence

**Fig. 3.**
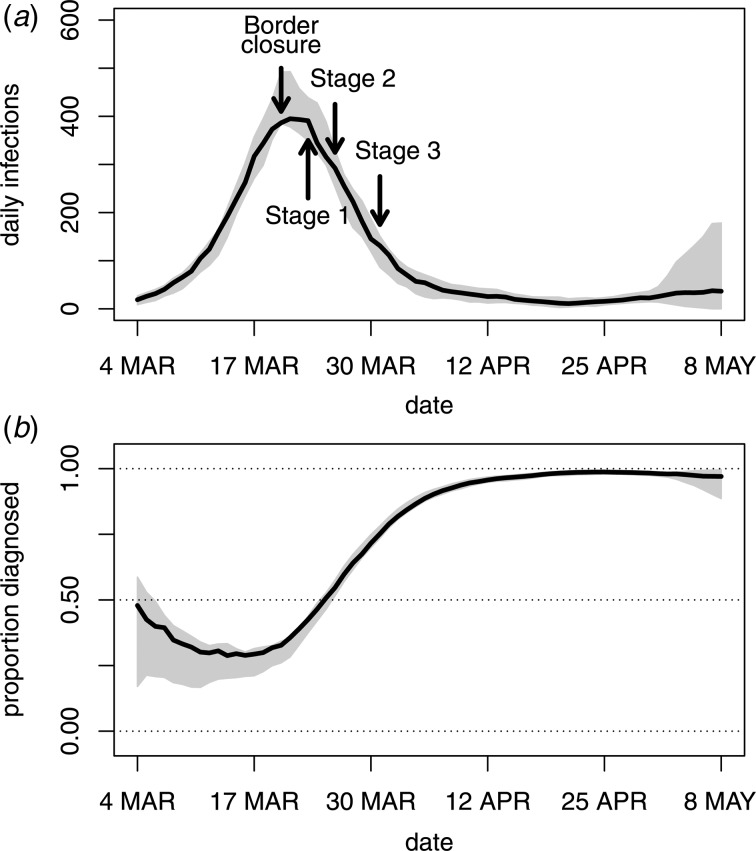
Back-projection estimates of COVID-19 infection incidence in Australia. Panel A plots the estimates of daily new infections and 95% bootstrap confidence intervals along with the timing of government restrictions. Panel B plots the ratio of diagnoses to infections and 95% bootstrap confidence intervals.

Figure 3 (panel A) shows the back-projection estimates of daily new infections. The estimates indicate a peak in the incidence of new infections at around 20 March. In contrast to the trend observed in the diagnosis data, border closures and Stage 1 restrictions were accompanied by a peak and decline in the incidence of new infections. To assess model fit and uncertainty, standardised Poisson residuals of observed daily diagnosis counts compared to fitted daily diagnosis counts were inspected and found to be centred around zero indicating good model fit. However, over-dispersion relative to the Poisson model was evident, with an estimated over-dispersion parameter of *σ*^2^ = 1.9. This over-dispersion was incorporated into the calculation of 95% confidence intervals displayed in Figure 3.

An important feature of Figure 3 (panel A) is the increase in variability of the back-projection estimates in the very recent past, which is a well-known property of back-projection analyses [6]. This reflects the fact that only a few diagnoses are the result of an infection in the very recent past, so there is less information about infection incidence in the last few days. As demonstrated by the widths of the confidence intervals, there is more information about earlier incidence and about the timing of peak incidence relative to the timing of government control measures.

### Undiagnosed infections

Due to the delay in diagnosing an infection, only a proportion of all past infections will be diagnosed at any given point in time. By subtracting the cumulative diagnoses from the cumulative infection estimates, it is possible to estimate the number of infections that remain undiagnosed. Figure 3 (panel B) provides the proportion of undiagnosed infections over time together with 95% confidence intervals. At the height of the epidemic, only one-quarter to one-half of all infections were diagnosed. Over time this proportion has increased and is approaching 1 as the incidence of new infections has become lower. Consistent with the confidence intervals in panel A of Figure 3, panel B displays some uncertainty about the undiagnosed proportion in the very recent past. Note that panel B of Figure 3 represents the proportion of all past infections that are undiagnosed, not the proportion of active infections. Furthermore, it is important to note that the undiagnosed infections do not include the undetected infections which are addressed in the next subsection.

### Sensitivity analyses

**Fig. 4.**
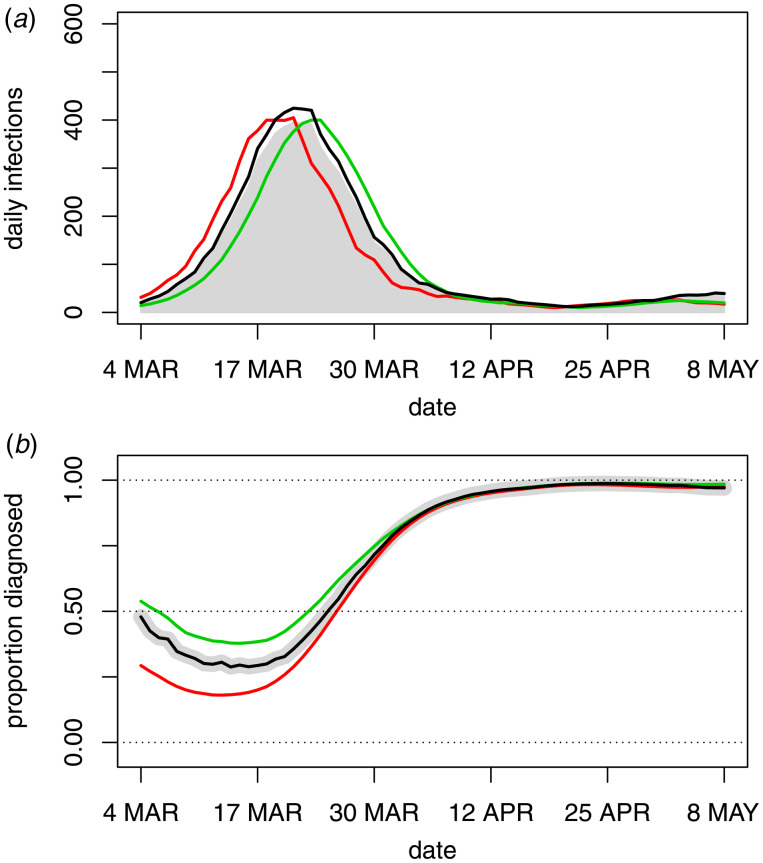
Sensitivity analyses for the main analyses presented in Figure 3. The main analyses (grey) are compared to an analysis with a long diagnosis distribution (red), short diagnosis distribution (green) and after adjusting for undetected infections (black).

Sensitivity analyses exploring the impact of different diagnosis distributions and adjustment for undetected infections, are displayed in Figure 4. The short diagnosis distribution (mean 5.1 days) shifted the incidence peak later while the long diagnosis distribution (mean 11.0 days) shifted it earlier. However, since these are extreme assumptions on the diagnosis distribution, there was not a high level of sensitivity and the broad conclusion that peak incidence occurred contemporaneously with the initial control measures is still applicable. Adjustment of incidence estimates for undetected infections raised the peak infection incidence but did not alter its position. Overall, given estimates of detection rates used by the Australian government [17, 18], adjustment for undetected infections had little effect on the analysis.

### Peak incidence and control measures

**Fig. 5.**
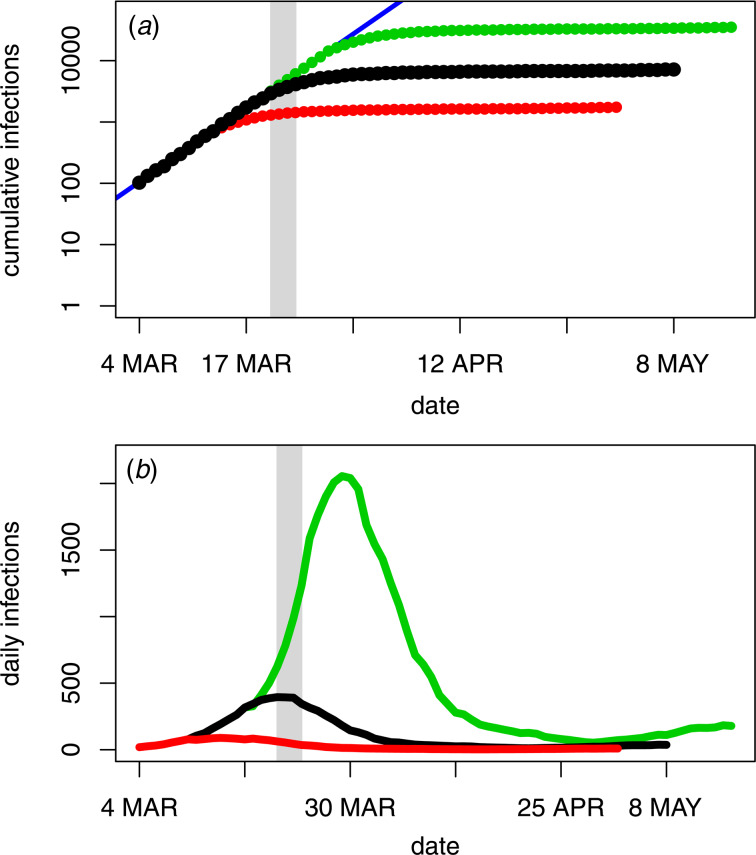
Back-projection estimates of COVID-19 infection numbers in Australia, showing transition from exponential to sub-exponential growth (black) relative to the timing of border closures and Stage 1 restrictions (20–23 March, shaded region). Also shown are the infection numbers obtained by shifting the transition to sub-exponential growth one week later (green) or one week earlier (red). Panel A displays cumulative infection estimates and panel B displays daily numbers of new infections.

As displayed in Figure 5 (panel A), the infection incidence estimates indicate that peak incidence and the initial government control measures were preceded by two weeks of exponential growth in cumulative infections (solid black points). This is reflected by a strongly linear trend on the logarithmic scale during these two weeks. Subsequently, the growth shifts from exponential to sub-exponential growth in cumulative infections. One possible explanation for this shift is the timing of the border closures and Stage 1 social distancing restrictions, reflected by the shaded region. By delaying the timing of this transition to sub-exponential growth by one week (green points) or moving it forward one week (red points), the effect of earlier or later control on the overall infection numbers can be investigated. Figure 5 (panel B) reflects the effect of these shifts on the estimates of infection incidence. Based on these estimates, one week later control would correspond with 35 050 infections (green line), an almost fivefold increase in total infections, whereas one week earlier control would correspond with only 1735 infections (red line).

### Further analyses

Consistent with Figure 2 (panel A), repeating the analyses on data from each of the three states with greater than 1000 diagnoses (New South Wales, Victoria and Queensland) yielded very similar trends to those displayed in Figure 5 for the national data (results not shown).

**Fig. 6.**
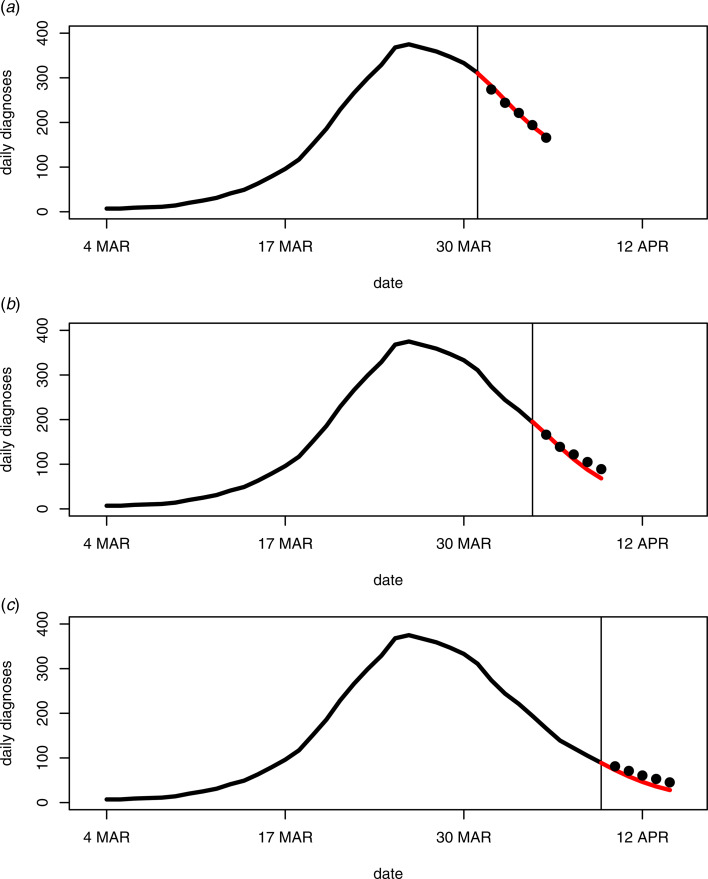
5-day forecasts (red lines) of daily diagnoses for the month of April based on back-projection analyses of data available prior to each day. The 5-day forecasts begin on 1 April (panel A), 5 April (panel B) and 10 April (panel C). Observed (smoothed) counts are shown as black lines up to the analysis day (vertical line) and as black points after the analysis day.

Also available are short-term projections of new daily diagnosis counts based on the fitted models from the back-projection analyses. Using the fundamental linear relationship between mean diagnoses and mean infections, as expressed in the Methods section, the fitted model can be used to calculate the mean number of diagnoses for a short period into the future. This is possible because the number of diagnoses in the next few days depends primarily on past infection incidence. Figure 6 presents 5-day forecasts for the month of April, subsequent to the date of the final government control measures (31 March). For these forecasts the back-projection analyses were repeated using data that were available prior to multiple days in April. The 5-day forecasts from these analyses were then compared with the actual (smoothed) counts that were subsequently observed. Figure 6 demonstrates the forecasts are a worthwhile predictive tool and a useful by-product of a back-projection analysis. Of course, other simple forecasting methods, such as extrapolation of a regression model fitted to the diagnosis counts, might also provide reasonable predictions. However, the forecasts in Figure 6 make use of the underlying infection incidence that drives future diagnoses, whereas simple extrapolation does not, which can lead to unreliable extrapolated diagnosis counts [6, Section 7.6].

## Discussion

Daily COVID-19 diagnosis counts are a valuable data resource providing information beyond the number of positive tests. Back-projection has been an important analytical method in prior epidemiological contexts, particularly HIV/AIDS, and has great potential for the analysis of COVID-19 data. This paper has presented a suite of analyses that can be undertaken on COVID-19 diagnosis data to assess infection incidence using the method of back-projection.

In Australia, the estimates of infection incidence show a peak around 20 March, less than two weeks after reaching 100 diagnoses. They also show that the initial key national government interventions, particularly border closures and Stage 1 social distancing restrictions, corresponded temporally with a peak and subsequent decline in infection incidence. Nonetheless, subsequent restriction stages are likely to have played a role in driving incidence even lower and preventing resurgence.

The estimates suggest that a one week delay in the timing of peak infection would have corresponded with a fivefold increase in the total number of people infected. Likewise, shifting the peak one week earlier would have yielded a decrease of similar magnitude in the total number of infections. Furthermore, the estimates suggest that at the height of the epidemic, the proportion of undiagnosed infections was half to three-quarters of the total infection pool. None of this information is directly discernible from inspection of the daily diagnosis counts, which continued to increase after the first stages of government control measures. This shows that the temporal association between control measures and the course of the epidemic may be quite different when the epidemic's extent is measured using estimated infection incidence rather than observed diagnosis counts. Back-projection estimates therefore provide useful additional information for assessing the effectiveness of control measures, as well as for understanding the evolution of infection incidence during the initial wave of infections and in the event of a second wave.

Back-projection may produce under-estimates of infection incidence if a proportion of infected individuals never have their infection diagnosed. This can be adjusted for by inflating the incidence estimates by a factor that depends on the detection proportion, which is an approach previously proposed in the context of HIV/AIDS [6, pp. 201–202]. Based on modelling used by the Australian government [17, 18], which estimates the detection proportion to be *P* = 0.93, this corresponds to a 7.5% inflation of the incidence estimates, which led to a slightly higher peak incidence when adjusting for undetected infections. Importantly, however, the shape of the infection incidence curve, and hence the timing of key features relative to the timing of government control measures, are unaffected by this under-estimation. Thus, the broad conclusions of the analysis are robust to this feature of the data. As the outbreak progresses, changes in the availability of testing may affect the proportion of undetected infections, which makes *P* time-dependent and affects the infection incidence curve. This could be accounted for by modelling a time-varying *P*, however, this is unlikely to be a major issue for the current analysis since testing was restricted to confirmatory case diagnosis.

Back-projection estimates are more uncertain in the recent past because few cases were infected in the recent past. Also, back-projection estimates may be sensitive to misspecification of the diagnosis distribution. For example, estimates of the incubation distribution may be based on data subject to retrospective ascertainment of infection dates and therefore subject to uncertainties and biases. Sensitivity analyses are therefore important in any back-projection analysis. For the current analysis, the primary qualitative conclusions concerning the pattern of infection incidence were robust to a range of diagnosis distribution assumptions.

As the COVID-19 pandemic evolves the diagnosis distribution may change, making it time-dependent. Such changes may result from introducing effective treatments, changes in the operational definition of disease, and changes in testing policy. Individual-specific factors may also affect the incubation distribution, such as age-dependencies. These complexities were present for HIV/AIDS, and generalised back-projection methodology has been developed [6, 7, 13]. Thus, while such influences would not have a substantive effect early in an outbreak, generalised methods may be required for COVID-19 in the longer term.

This paper has applied back-projection to daily diagnosis counts, however, it could also potentially be applied to routinely reported daily mortality counts. This would require detailed information on the probability distribution of the time from infection to death. Since death from COVID-19 infection is likely to have less under-reporting than case diagnosis, death data would have the advantage of being more complete. Nonetheless, only a small proportion of infections result in death, so back-projection estimates from death data would need to be adjusted using external information about the mortality rate. Another issue is that death data may contain substantially less information than diagnosis data. For example, in Australia where approximately 100 deaths have occurred, such analyses would not be highly informative. Nonetheless, for high mortality countries back-projection of death counts may be informative about past infection incidence and future mortality.

An important line of future research is to explore the use of back-projection estimates of detectable infection incidence for calibrating transmission dynamics models. Such models make critical parameter assumptions which should lead the models to track the infection incidence estimated by back-projection. Such an approach to transmission model calibration was used effectively in the HIV/AIDS context and may have applications for COVID-19 modelling [9, 21]. This should be a high priority for COVID-19 epidemiological research, and would require close collaboration between modellers and statisticians.

Future work should also focus on developing automated analytics systems for continuous updating of estimates and projections as the outbreak evolves on a daily basis. Such analyses would add value to the descriptive dashboards that have proliferated in the wake of the highly successful Johns Hopkins University Coronavirus Visual Dashboard [1, 2]. Analytics software for serial updating of estimates of basic reproduction numbers has previously been advocated [22]. However, currently there is only limited implementation of back-projection methodology for COVID-19, through the Coronavirus 10-day Forecast resource from The University of Melbourne [4] and the epiforecasts resource from the London School of Hygiene and Tropical Medicine [23]. More expansive use of back-projection would allow continuous monitoring for resource planning and identification of key milestones, such as an infection incidence peak or a resurgence in infections as control measures are eased.
